# Supplementary material for: Abrupt reduction in shipping emission as an inadvertent geoengineering termination shock produces substantial radiative warming
Source: Commun Earth Environ. 2024 May 30;5(1):281. doi: 10.1038/s43247-024-01442-3 (PMC11139642; doi:10.1038/s43247-024-01442-3)
Supplement: Supplementary file 2 — Supplemental Material [file 43247_2024_1442_MOESM2_ESM.pdf]

Supporting Online Material

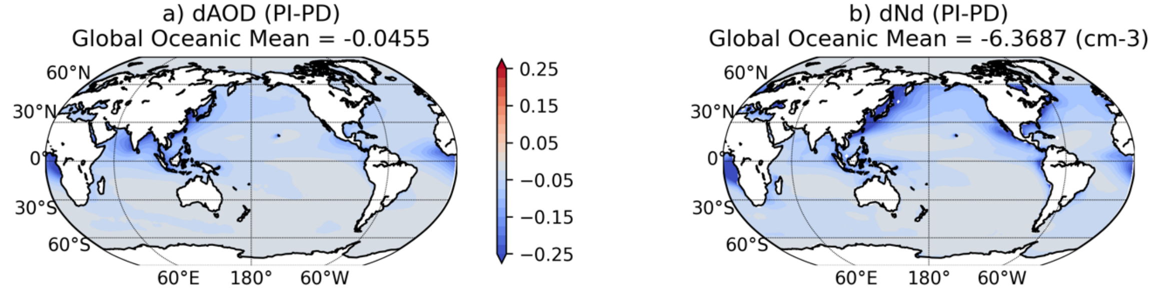

Figure S2: Total AOD change (a) and N_d change (b) between pre-industry and present day. This is based on results from Bellouin et al. (2020)[2] and Toll et al. (2019)[16]. We exclude data over land and calculate the mean value over the ocean. It is worth pointing out that estimates on the AOD changes between present day and pre-industry have uncertainties due to methodological and modeling differences. We use this estimate because ΔN_d between present day and pre-industry is also estimated using this data source.

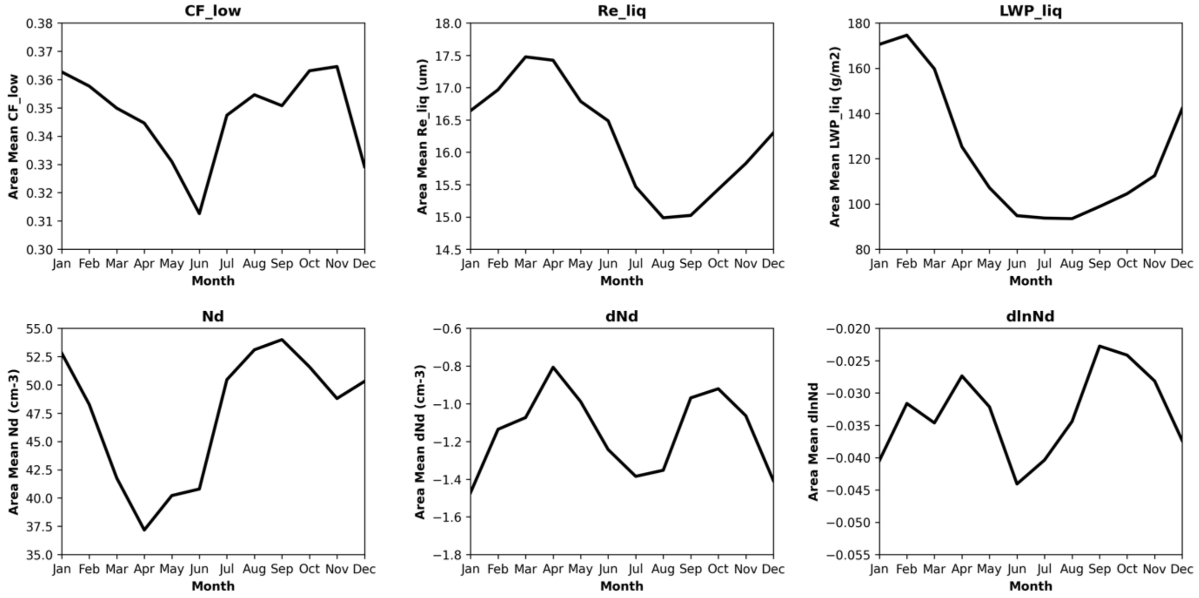

Figure S3: Seasonal cycle of low cloud fraction, R_e, LWP, N_d, ΔN_d, and ΔlnN_d averaged over the North Atlantic. These panels illustrate the substantial seasonal variations in different quantities that drives the seasonal changes in IMO2020 induced radiative forcing together with solar insolation.

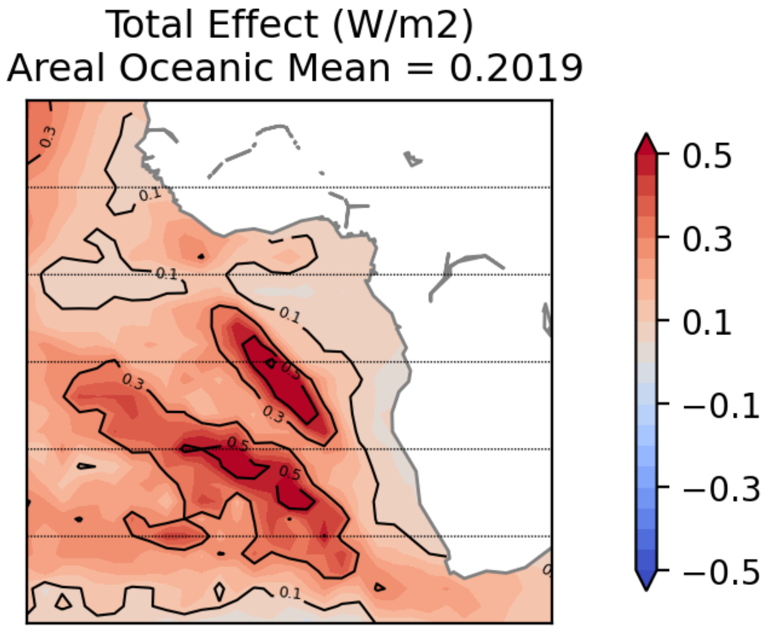

Figure S4: Total aerosol indirect forcing in the Southeast Atlantic region due to IMO2020. In the core shipping lane, where the maximum warming is estimated, the peak value is around 0.5 Wm^{-2} in excellent agreement with Diamond (2023)[19] in the same area. The core shipping lane refers to the northern peak in this plot because it contains the strongest ship-emission [19].

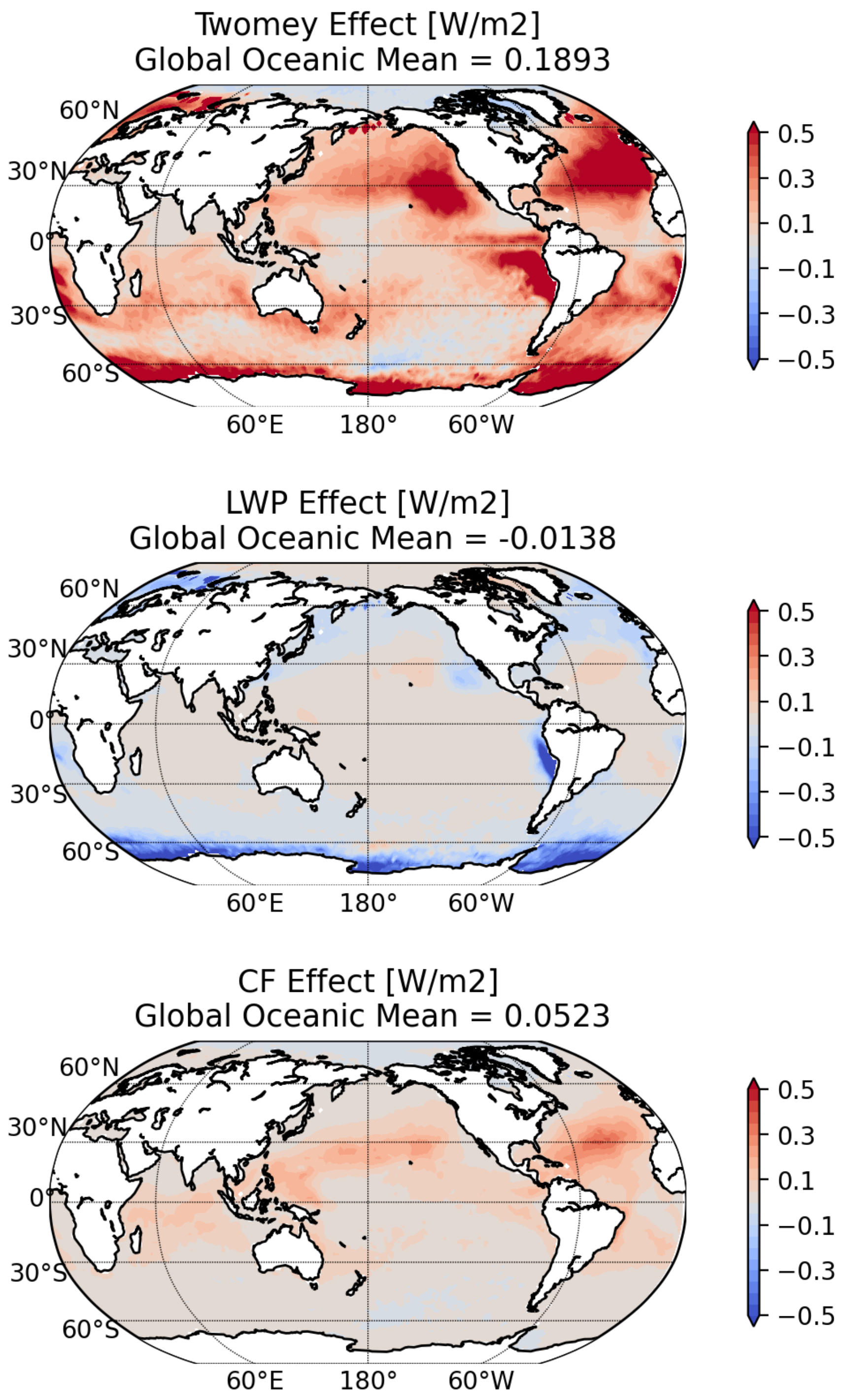

Figure S5: The forcing from effects of both COVID induced and IMO related emission decrease. The total forcing exceeds 0.25Wm^{-2}, doubling the impact of IMO alone, which means the climate experienced a stronger shock in 2020 due to the double effects of COVID and IMO2020.
