# Supplementary material for: Abrupt reduction in shipping emission as an inadvertent geoengineering termination shock produces substantial radiative warming
Source: Commun Earth Environ. 2024 May 30;5(1):281. doi: 10.1038/s43247-024-01442-3 (PMC11139642; doi:10.1038/s43247-024-01442-3)
Supplement: Supplementary file 3 — Supplemental figure and table [file 43247_2024_1442_MOESM3_ESM.docx]

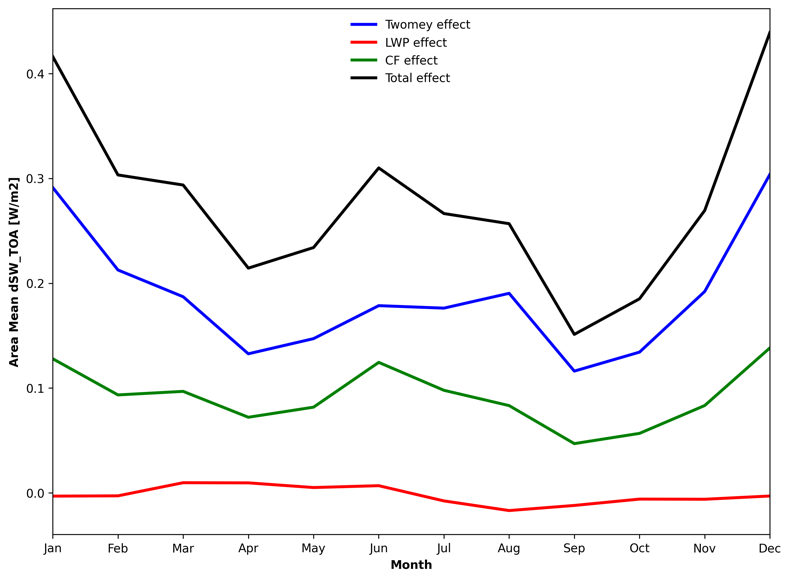


**Figure S1: Seasonal variations of areal mean radiative forcing over the North Atlantic if a seasonally invariant incoming solar isolation (annual mean at each grid) is used. The peak in the summertime in Figure 3 is replaced by a wintertime peak.**

**Table S1: A summary of SO_2_ emissions under different scenarios**

| Experiment^1^ | Emi-an^2^ | Emi-shp | Emi-vol | Emi-bio | Emi-bb |
| --- | --- | --- | --- | --- | --- |
| shpallBAU | 41.3 TgS | 5.3 TgS | 11.4 (TgS) | 15.3TgS | 1.9 (TgS) |
| Shp1p7BAU | 36.8 | 0.8 |  |  |  |
| Shp0BAU | 36 | 0 |  |  |  |
| shpallCovid | 36.4 | 4.4 |  |  |  |
| Shp1p7Covid | 32.6 | 0.7 |  |  |  |
| Shp0Covid | 32 | 0 |  |  |  |
